# The Distribution between the Dissolved and the Particulate Forms of 49 Metals across the Tigris River, Baghdad, Iraq

**DOI:** 10.1100/2012/246059

**Published:** 2012-11-28

**Authors:** Samera Hussein Hamad, James Jay Schauer, Martin Merrill Shafer, Esam Abed Al-Raheem, Hyder Satar

**Affiliations:** ^1^Environmental Chemistry and Technology Program, University of Wisconsin-Madison, 660 North Park Street, Madison, WI 53706, USA; ^2^Trace Elements Research Laboratory, Wisconsin State Laboratory of Hygiene, 2601 Agricultural Drive, Madison, WI 53718, USA; ^3^Hazmat Office, Iraqi Ministry of Science and Technology, Al-Jadriya District, Baghdad, Iraq

## Abstract

The distribution of dissolved and particulate forms of 49 elements was investigated along transect of the Tigris River (one of the major rivers of the world) within Baghdad city and in its major tributary (Diyala River) from 11 to 28 July 2011. SF-ICP-MS was used to measure total and filterable elements at 17 locations along the Tigris River transect, two samples from the Diyala River, and in one sample from the confluence of the two rivers. The calculated particulate forms were used to determine the particle-partition coefficients of the metals. No major changes in the elements concentrations down the river transect. Dissolved phases dominated the physical speciation of many metals (e.g., As, Mo, and Pt) in the Tigris River, while Al, Fe, Pb, Th, and Ti were exhibiting high particulate fractions, with a trend of particle partition coefficients of [Ti(40) > Th(35) > Fe(15) > Al(13) > Pb(4.5)] ∗ 10^6^ L/kg. Particulate forms of all metals exhibited high concentrations in the Diyala River, though the partition coefficients were low due to high TSS (~270 mg/L). A comparison of Tigris with the major rivers of the world showed that Tigris quality in Baghdad is comparable to Seine River quality in Paris.

## 1. Introduction

 River waters are the most important fresh water resource, as ancient to present day civilizations have flourished along their banks. For a long time, rivers have been used for cleaning and disposal purposes, and as a result they are subjected to large loads of wastes from industries, domestic sewage, and agriculture [1]. Urban rivers are among the most prone to pollution due to their close proximity to many pollution sources like domestic wastewaters, industrial effluents, and solid-waste disposal sites [2]. People in urban areas depend totally on the rivers as a main source of drinking water; thus, monitoring river waters is important not only to evaluate pollution sources but also to ensure an efficient management of water resources to protect aquatic life [3]. Trace and heavy metals are the most common environmental pollutants, and their occurrence in waters and biota indicates the presence of natural or anthropogenic sources [4], which can have a serious impact on plants and animal life [5]. Some metals are fatal to organisms at certain levels [6], while others such as As, Pb, and Cd exhibit extreme toxicity even at trace levels [7]. However, the rates of trace and heavy metals reactivity and their distribution vary between rivers due to variable environmental factors such as transport processes, hydrodynamic residence time, and mixing patterns. Hence, there is no universal pattern of trace metals behaviors in estuaries [8]. The distribution process of heavy and trace metals is a key issue as it determines the mobility and toxicity of the metals within estuaries and coastal oceans [9]. Many studies have shown the adsorption/desorption reactions of metals in waters depend on a number of factors including pH, salinity, redox conditions, temperature, and the composition of suspended particulate matters (SPMs). The distribution coefficient (*K*_*d*_ or *K*_*p*_) is the most common means of describing the solid-dissolved form of metals in water [10].

 In this study we focus on the solid/dissolved distribution of heavy and trace metals in the Tigris River, the largest river in Iraq with 1,450 km of its total length of 1,900 km flowing through Iraq. This river arises from the Taurus Mountains south of Turkey with 450 km flowing through Turkish and Syrian territory before entering Iraq [11]. The Tigris passes through two large cities before it enters Baghdad, “Diyarbakir” south of Turkey and “Mosul” in northern Iraq. These cities are large centers of industry and agriculture and have been adding significant contamination to the Tigris River [12]. In addition, the Tigris River in Iraq has been prone to serious contamination for a long time due to continuous armed conflicts during 1980, 1991, 1998 and 2003 and the ensuing turmoil. A substantial amount of contamination has been added to the river by industrial wastes, oil derivatives, military wastes, spilling, burning, and looting of toxic chemicals from industrial complexes [13]. The Tigris River is the only river which passes through Baghdad “the largest city in Iraq with a population of about 7,216,000,” and the only source of water in the city. Results from a case study of the Tigris River showed a significant amount of pollution has been added to the river after passing through Baghdad city [14]. 

Although the Tigris River is the most important water source in Iraq, obtaining and monitoring the necessary quality and quantity of this river represents a significant problem for Iraq [15]. Studies that have been conducted to evaluate the quality and quantity of the Tigris are quite simple and limited due to a lack of knowledge and supplies available in Iraq which is a result of sanctions imposed on Iraq for many years. Some studies have focused on total concentrations of a narrow suite of metals [16–18] while other studies have evaluated the Tigris quality and quantity by mathematical models [19]. No data have been reported for the distribution of trace and heavy metals in the Tigris River “the largest river in Iraq and one of the major rivers of the world” [20]. 

 In this study we measured the concentration of 49 elements in the Tigris River within the Baghdad borders, where many sewages, hospitals, and industries dump their refuse. Magnetic sector-inductively coupled plasma/mass spectrometer (ICP-MS) was used to analyze 49 “trace and heavy” metals across the Tigris River in Baghdad. Two methods were used to study the distribution of metals across the river. The first is the measurement method in which total and dissolved metal concentrations were measured using SF-ICP-MS; the second method is the calculation of the particulate-metals form and partition coefficients of the assessed metals. The two methods were applied to 17 samples sites along the Tigris River within Baghdad city, two samples sites from Diyala River, and one sample from the confluence of the two rivers south of Baghdad.

## 2. Field Sampling

**Figure 1:**
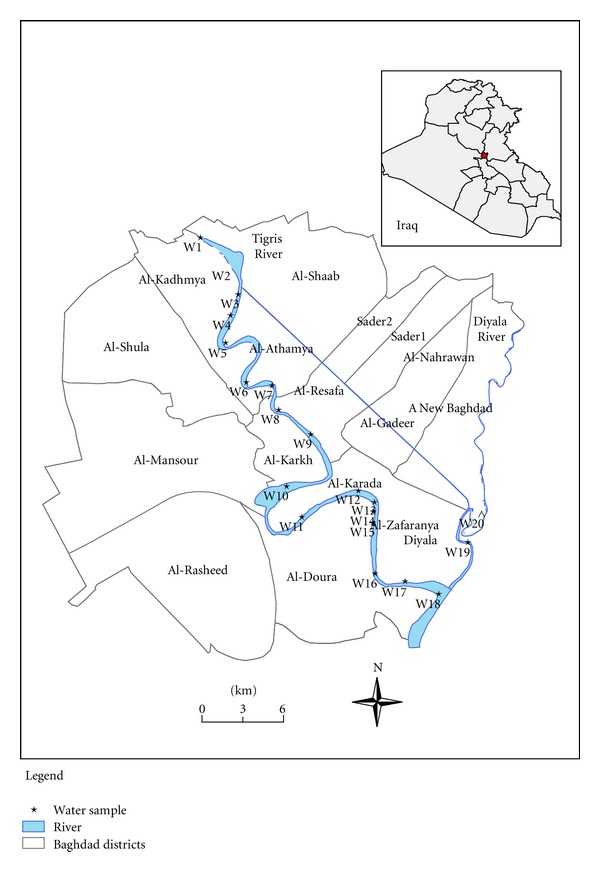
Study area shows 17 sampling sites along Tigris River and two samples from Diyala River with the meeting point (W18) down of the city.

Twenty water samples were collected from 11 to 28 July 2011. Seventeen of which were sampled on Tigris River within Baghdad city, two samples were samples from the Diyala River, one upstream of the confluence the Tigris, and one sample was collected south of Baghdad city Figure 1. Water samples were collected in acid-washed 500 mL Teflon bottles opened and closed under water to avoid surface microlayer contamination. The sampling team employed the “clean-hands/dirty-hands” technique for sample collection. Samples were collected approximately 2-3 meters from shore, and the sample bottle was immediately placed in the inner polyethylene (LDPE) zip-lock bag by the “clean-hands” person and then double bagged by the “dirty-hand” person. Samples were kept cool using a cooler box. pH, conductivity, and water temperature were measured in the field using Oakton-pH meter and Oakton-conductivity meter. All sampling sites coordinates were recorded using a GARMIN-GPS.

 Field analysis typically started within 2-3 hours of samples collection. Approximately 55 mL of the “unfiltered” water was poured from each 500 mL Teflon collection bottle into a 60 mL acid-cleaned preweighed LDPE bottles and then kept frozen until the time of analysis. Filtered water was prepared by filtering 50 mL of the unfiltered water using a vacuum in an all plastic filtration system which collected the filtrate directly into 60 mL preweighed, acid-washed LDPE bottles. The filtered water samples were immediately frozen. The polyethersulfone filters were 47 mm in diameter with a 0.45 *μ*m pore size. Particles were separated/collected on the preweighed 0.45 *μ*m filters. Filters were immediately placed into individual plastic petri dishes, wrapped in Teflon tape, and kept frozen. All the samples (filtered and unfiltered water, and the filters) were shipped on blue ice to the trace element laboratory in Madison, WI, USA, for analysis.

## 3. Materials and Methods

### 3.1. Samples Preparation

Forty-nine elements were analyzed for each water sample collected from the Tigris River, Diyala River, and the site at the meeting point of the two rivers. The metals were differentiated between operationally defined dissolved and particulate forms depending on whether or not they passed through the 0.45 *μ*m filters. Filtered samples were acidified to 2% HNO_3_ (Baker Ultrex Grade), while the unfiltered water samples were digested by acidifying the samples to 7%(v/v) with Ultrex HNO_3_ and then heated in the original (60 mL) Teflon bottle for 24 h at 55°C.

### 3.2. Trace Metal Analytical Procedure

 Sample preparation and SF-ICP-MS analysis were performed in HEPA filtered air within the trace metal clean lab. The digested “unfiltered” samples were used to estimate the total metals concentrations. Filtered samples, which were acidified to 2% (v/v) using high-purity 16N nitric acid, were analyzed for dissolved metals. Samples were transferred to autosampler vials under a clean hood by clean room-garmented personnel. Forty-nine elements were quantified by magnetic-sector ICP/MS (Thermo Finnigan Element 2). This instrument is capable of quantifying trace elements at sub ng/L levels, as well as major elements (at higher mass resolution) in aqueous samples. Multielement external standards were used to calibrate the instrument over the expected sample concentration ranges. And 2 *μ*g/L mix of Ga, In, and Bi in 2% HNO_3_ was used as an internal standard, which was added on-line via a mixing coil before the sample stream enters the FEP nebulizer. Strict QA protocols were followed to enable accurate and precise quantification at trace levels. This included acquisition of multiple isotopes, extensive blank measurements, duplicate sample analysis, matrix spike recoveries, and frequent analysis of a standard reference material (SRM: SLRS-5). Additional information about the laboratory analytical protocols used in this study can be reviewed in the following studies [21]. Samples were analyzed for forty-nine elements (Table 1 in the Supplementary Materials available online at doi: 10.1100/2012/246059). The mean analytical instrument precision for total trace elements at typical environmental levels was 4.8% and for the major metals was 1.6%. The mean analytical precision of the trace elements in the filtered samples was 10% and 2.4% for the major elements. Filters were dried for a period of 36 hours, and then reweighed at two separate dates on a microbalance to a significant figure of 1 *μ*g to quantitate dry particle weight. 

## 4. Result and Discussion

 The data presented and discussed below is from the seventeen water samples collected from a transect of the Tigris River; two samples from the Diyala River taken close to the wastewater treatment plant and one sample taken from the meeting point of the two rivers south of Baghdad (Figure 1). Sample location W3 has been excluded from all data plots in this study due to very high levels of particulate metals created from a treatment plant discharge north of Baghdad city, but its value has been taken into account to calculate the average concentrations in the Tigris River.

### 4.1. Physical Properties

**Figure 2:**
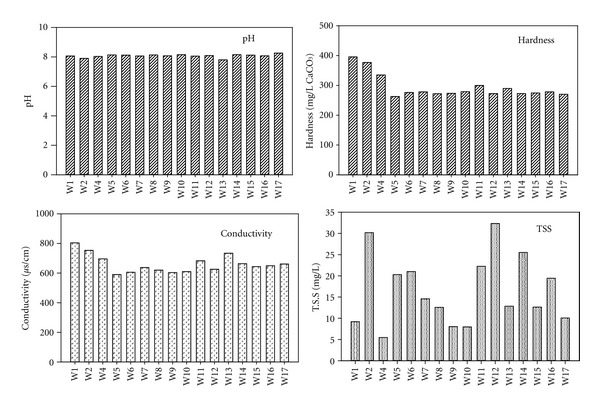
pH, hardness, conductivity, and total suspended solid (TSS) across Tigris River.

**Figure 3:**
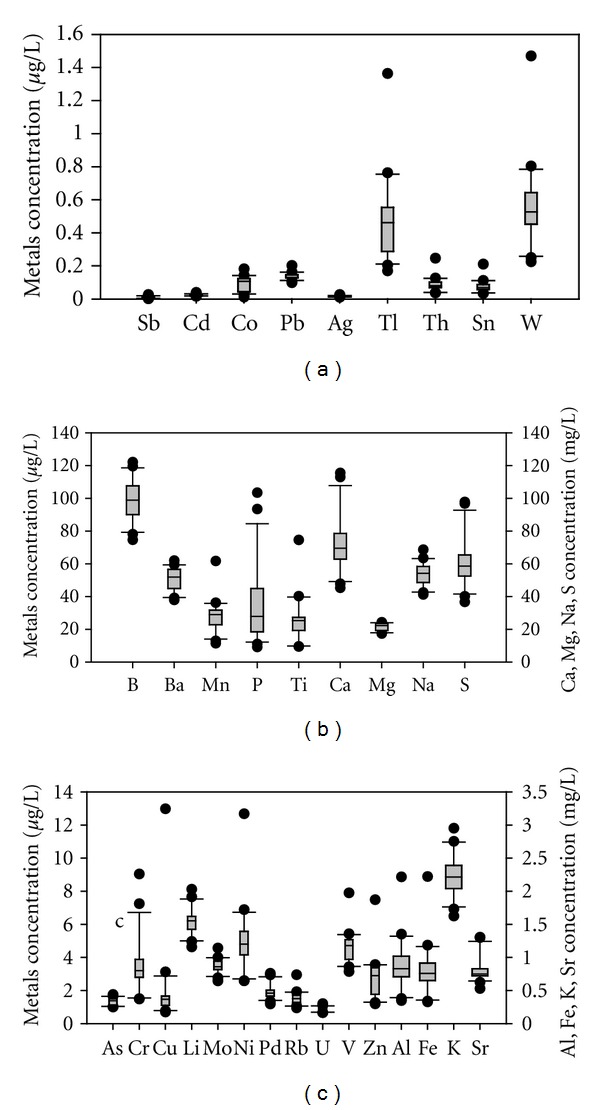
Box plots of the metals across Tigris River: (a) all the metals are in *μ*g/L; (b) Ca, Mg, Na, and S are in mg/L; (c) Al, Fe, K, and Sr are in mg/L.

 Physical properties were measured across the Tigris River. In general, pH values are almost uniform across the Tigris River. For hardness, just a few sites W1: 395 mg/L CaCO_3_, W2: 376 mg/L CaCO_3_, W4: 334 mg/L CaCO_3_, exhibited values outside the nearly stable hardness concentrations in the river. Sp.C also saw little change for all Tigris sampling sites. There were large fluctuations for TSS/SPM across the Tigris. High concentrations of TSS were present at sites close to discharges of industrial facilities (sites W11, W12, and W14) and intensive agricultural application (site W2).  Figure 2 shows the distribution of these field tests across the Tigris in July 2011. The average discharge of the river was approximately 596 m^3^/sec (according to AL-Sarai Gauging Station Report Baghdad/Iraq/2011), and the average water temperature was 30.1°C. The Tigris River pH ranged from 7.8 to 8.3, while the Diyala River was 7.4. pH levels are all within the IDWQS (6.5–8.5) and the WHO & USEPA allowable limits (6.5–8.5). Hardness in the Tigris ranged between 262 and 395 mg/L CaCO_3_ and 908 mg/L CaCO_3_ in the Diyala River. Hardness levels in the Tigris River exceeded the IDWQS permissible limits. A previous study of the Tigris River has shown there is no difference of hardness concentration between raw and treated water “for drinking,” as it stayed between 215 and 465 mg/L CaCO_3_ from Nov. 2005 to Oct. 2006 [26]. However, there are no health-based guidelines proposed for hardness in drinking water [27]. The Tigris River Sp.C ranged from 590 to 804 *μ*S/cm, while the Diyala River Sp.C ranges were 2400–2630 *μ*S/cm. At the sampling site where the Tigris and Diyala meet, the Sp.C was 2600 *μ*s/cm—indicating that the sample was largely influenced by the Diyala. The conductivity of the Tigris River was within the IDWQS but exceeded the USEPA allowable limit [28], while the conductivity for both the Diyala River and the meeting point exceeded the IDWQS and USEPA allowable limits. The Tigris is characterized by relatively high concentration of Ca, Mg, and S (Figure 3(b)), the reason of the high hardness in the river. The average TSS for the Tigris was 15.9 mg/L, and 270 mg/L in the Diyala River. All TSS measurements (see Figure 2) fall within the levels reported in previous studies [29–31].

### 4.2. Total Metals Concentrations

 Total metals concentrations of 49 metals in the Tigris, and Diyala Rivers were measured in unfiltered and digested water using ICP/MS. Table 1 in the supplementary material shows the mean concentrations (and standard deviations) of all metals at the 17 Tigris and the two Diyala River sites near treatment plant wastewater discharge south of Baghdad city. All metals concentrations in the Diyala River were substantially higher than the Tigris River. In this paper we focus on the total concentration metals that have been shown to have negative health effects [32].  Table 1 shows the minimum and maximum concentrations of toxic metals in the Tigris River compared with the permissible levels established by WHO and the USEPA [23–25, 33] and the WA [29–31]. Most metal concentrations are within permissible levels (in *μ*g/L) except Al: 347–2,220, Fe: 326–2,220, Sn: 0.012–0.181, Sr: 528–1,304 (Figure 3). There is little or no indication that orally ingested aluminum at these levels is toxic to humans, specifically for the neurological system [22]. Similarly there are no known health concerns with regard to iron at these levels in drinking water [34]. Tin (Sn) levels in rivers are generally less than 0.005 *μ*g/L, but the use of organotin biocides can produce higher concentrations [35]. In the Tigris River Sn levels are somewhat higher than the permissible levels (Table 1; Figure 3(a)); however, there is limited data to indicate adverse effects in humans associated with chronic exposure to tin [25]. Elevated strontium concentrations in drinking water (>6,000 *μ*g/L) may result in dental problems [36]. Most of the metals concentrations in the Diyala River are higher compared to the Tigris and exceed WHO and USEPA permissible limits. The higher metal concentrations in the Diyala River are likely due to the discharge from the wastewater treatment located near this sampling site. 

Few studies have reported REE concentrations in rivers and other surface waters [37]. Figure 1 in the supplementary material section shows boxplots of the total concentrations of REE along with some platinum group metals in the Tigris and Diyala Rivers. The concentrations of REE in the Tigris generally fall in the range of REE concentrations reported in a previous study [38], but are higher compared to other studies [39]. All the Diyala River REE concentrations exceeded the concentrations reported in the above-mentioned studies (Table 1 and Figure 1 in supplemental materials). 

### 4.3. Distribution of Metals between SPM and the Dissolved Phases

 Filtered water (<0.45 *μ*m) was used as a proxy for the dissolved metals concentrations in the Tigris, and Diyala Rivers. Metals in the filterable form could be “free” aqueous metal ions, inorganic complexes, and/or bound to organic ligands. The particulate metal phase is calculated by subtracting the dissolved metal concentrations from the total metals concentrations. SPM is a complex of particle components that may include resuspended sediment, autochthonous biological material, and allochthonous soils, to which trace elements may associate [40]. 

**Figure 4:**
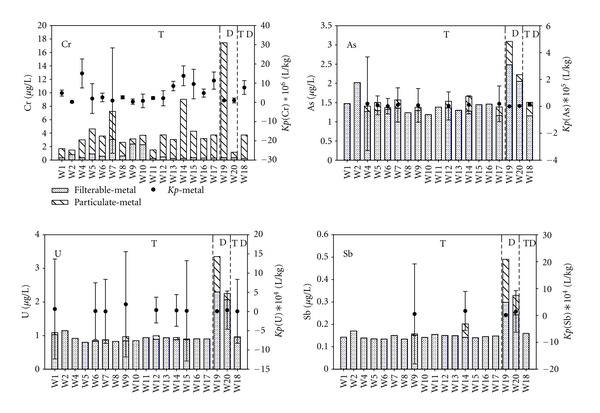
Distribution of Cr, As, U, and Sb with the partition coefficients in Tigris (T), Diyala (D), and the meeting point (TD) of the two rivers.

**Figure 5:**
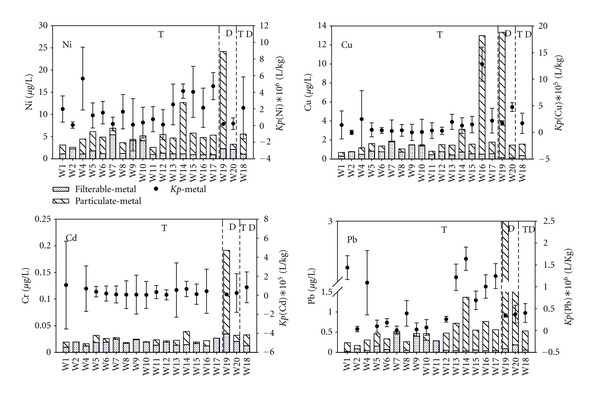
Distribution of Ni, Cu, Cd, and Pb with the partition coefficients in Tigris (T), Diyala (D), and the meeting point (TD) of the two rivers.

We focus our discussion on selected metals (As, Cd, Cr, Cu, Ni, Pb, Sb, and U), which are of considerable interest because of their potential toxic and carcinogenic properties. The physiological behavior of these elements is known to depend on their oxidation state and chemical form. Figures 4 and 5 show the concentrations of dissolved and particulate forms of metals in the Tigris River transect (represented by 17 sites), at two sites in the Diyala River and the site located at the meeting point of the two rivers. In general chromium concentrations in the Tigris were within the WHO and USEPA allowable limits (Table 1, Figure 4). Particulate phase chromium is the dominant physical form. The dominant dissolved chemical species of chromium in oxic natural waters are Cr (III) in the form of Cr(OH)_2_^+^·4H_2_O and Cr(VI) in the form CrO_4_^−2^ [41]. Chromium (III) species typically have low solubility and may readily sorb to SPM, potentially removing it from the water column by settling. Cr(VI) species are more soluble, but can sorb to manganese/iron hydrous oxides. In the Tigris, there is little significant variation in the chromium level except for the two sites W7 and W14. Two sites, both located immediately downstream of significant discharges, exhibit higher chromium levels (site W14, downstream of the heavily industrialized site W13, and site W19 in the Diyala River which was immediately downstream from the discharge of the wastewater of the treatment plant). In contrast to chromium, the dissolved phases dominate the physical speciation of arsenic (Figure 4). This reflects the oxyanion chemical speciation of arsenate and arsenite. These inorganic species are more toxic than both organoarsenic species and particulate forms [42]. There was no major variation in arsenic levels across the Tigris River, with an average filterable concentration of 0.296 *μ*g/L. Particle-phase arsenic was elevated in the Diyala River (0.392 *μ*g/L). Arsenic concentrations in the Tigris River (Table 1) and the Diyala River are within the permissible levels (Table 2).

Concentrations of total uranium are nearly uniform across the Tigris River transect (Figure 4) with an average concentration of 0.89 *μ*g/L (Table 2). Diyala River Uranium levels were twice that of the Tigris River (Table 2, Figure 4), which might be related to the sabotage of the Iraqi Atomic Energy Agency (south of Baghdad) after 2003 conflict, and the uranium drums that have been stolen from the agency at that time [13]. The dominant chemical species of uranium (uranyl carbonate UO_2_(CO_3_)_3_^4−^ [43] (VI)) is very soluble in oxygenated water which may explain the near complete absence of uranium bound particulate species (Figure 4). Uranium levels across the Tigris River are within the WHO and USEPA allowable limits (Table 1). Total antimony concentrations exhibit a uniform trend for all the sites across the Tigris with levels below the WHO and USEPA allowable limits (Table 1). Antimony concentrations were elevated in the Diyala River and exceeded the WHO and USEPA permissible levels (Table 2). Similar to uranium, particulate forms of antimony were undetectable across the Tigris except at sites W9 and W14 where Sb concentrations were 0.006 *μ*g/L and 0.06 *μ*g/L, respectively. Site W9 is located 2,980 m downstream of a hospital discharge; and site W14 is immediately downriver from a heavily industrialized discharge site in the Al-Zafarania district. Antimony can be found in natural waters in two oxidation states, the pentavalent form Sb(V) and the trivalent species Sb(III) which is reported to be more toxic than Sb(V) [44]. The particulate form of antimony was higher at sites W19 and W20 which are close to a wastewater treatment plant discharge (Figure 4). 

 Total nickel concentrations in both the Tigris and Diyala rivers are within the WHO and USEPA permissible levels, but exceed the WA [30, 31] (Tables 1 and 2).  Figure 5 shows that while significant concentrations of both dissolved and particulate phase nickel are present, the dissolved form is typically greater in both rivers (Tigris and Diyala). Higher dissolved nickel is observed at site W7 (5.4 *μ*g/L) which is located downriver of a hospital discharge, and particulate phases are elevated at site W14 (11.6 *μ*g/L) and Diyala River site W19 (216 *μ*g/L). The higher concentrations of particulate Ni are most like from the industrial discharges (W14) and the treatment plant (W19). Measured copper concentrations in the Tigris and Diyala Rivers are significantly below the WHO and USEPA permissible levels. Similar to nickel, higher concentrations of particulate copper were observed at sites W16 (immediately downriver of a beverage factory) and W19 the site after the discharge of wastewater treatment plant on the Diyala River (Figure 5). Both copper and Nickel partition strongly to organic matter [45] and the high particulate levels may be related to direct or indirect partitioning of Ni to SPM. 

Cadmium is predominantly in the dissolved form along the Tigris transect (Figure 5), and concentrations are all within the WHO and USEPA permissible levels (Table 1); Cd concentrations varied little across the Tigris transect, while the major exception is at site W19 in the Diyala River after the treatment plant discharge where the particulate phase is dominant, reflecting the impact of the high SPM concentration at this site. Lead strongly partitions to particles [46], and this is clearly evident in both the Tigris and Diyala river samples (Figure 5), where the Pb is predominately in the particulate phase. Dissolved Pb levels were generally quite low along the Tigris transect (average = 0.379 *μ*g/L), while the high Pb concentration of 15.5 *μ*g/L at site W19 is most likely impacted from the high SPM created from the treatment plant discharge. 

In the supplemental materials data is presented (Table 1 and Figures  2,  3, and  4) for a large number of additional metals. This data is briefly discussed here. In general, Na, and K do not exhibit major concentrations changes along the Tigris River, while Al and P do exhibit concentration changes (Figure 2). Thorium, Tl, and W have different concentrations along the Tigris depending on the site location, but Mo is almost uniform across the main river (Figure 3). Manganese and V concentrations fluctuated, but within a relatively narrow range, along the Tigris River. A major increase in Sn concentration at site W7 was measured, and Ag concentrations exhibited significant variation across the main river (Figure 4).  Figure 2 presents data for Na, K, P, and Al in the soluble and particulate forms across the Tigris transect. Sodium as expected is almost entirely in the dissolved form. The Diyala River has a sodium level 7-times higher than the Tigris. The same trends are observed for potassium with only minor amounts of particulate K at the Tigris (445 *μ*g/L) and Diyala River (1,370 *μ*g/L), (Figure 2 in supplemental materials). Phosphorus levels were generally low in the Tigris. The total phosphorus concentration averaged 35.7 *μ*g/L, of which 14.2 *μ*g/L was in the dissolved form and 21.5 *μ*g/L was in the particulate form. The Diyala River total phosphorus concentration was 2,140 *μ*g/L (Table 2 in supplemental materials), all of which was in the dissolved form (Figure 2 in supplemental materials). It is highly probable the high dissolved-P levels at sites W19 and W20 are the result of the wastewater treatment plant discharge into the Diyala River; sewage is a major source of dissolved phosphorus in river water [47]. Relatively high total aluminum concentrations are observed in the Tigris (Table 1 and Figure 2 in supplemental materials). The physical speciation of Al was predominately in the particulate phase in most sites across the river (W7, W9, and W10 were exceptions). There are several hospitals and domestic discharges between sites W9 and W10 along the Tigris, and site W7 is located downstream of a hospital discharge. However, it is unlikely the discharges are directly influencing the Al levels and partitioning. The aluminum levels are higher than EPA, WHO recommendations, and at these levels, aluminum could affect the human neurological system [22, 48]. 

Figure 3 in the supplemental materials shows the soluble and particulate concentrations of Th, Tl, Mo, and W levels along the Tigris transect and in the Diyala samples. The transect pattern and particle partitioning of Th is nearly identical to that observed for aluminum, suggesting common vectors. Thorium (Th) total concentrations are in the range discussed in previous studies [49], and the physical speciation is predominately in the particulate form which is consistent with known extreme particle reactivity [49]. Thorium is a decay product of U, and in oxic environments such as river water, U is quite soluble (Figure 2) relative to Th; as a result U is leached out of the solid phase (e.g., uranyl carbonate is the dissolved form), whereas Th would remain in the solid phase (Figure 3, supplemental material). The phase distribution of thallium shows significant levels of both soluble and particulate forms. Soluble levels (likely the Tl(I) species) are quite uniform along the Tigris transect [50]. Site W14 which is located downriver of the heavily industrialized area had a high particulate-Tl concentration of 0.009 *μ*g/L, as did the Diyala River site W19 (0.014 *μ*g/L) (Figure 3, supplemental material). Molybdenum tends to be in the dissolved form with little or no variation in total concentration along the Tigris transect. The one exception is the high particulate levels in the Diyala River (W19, W20). All of the Mo concentrations are within the WHO and USEPA permissible levels (Table 1). Tungsten's particle partitioning (Figure 3, supplemental material) appears more variable than other metals examined. The dissolved form is dominant at many sites, while tungsten at other sites along the Tigris (W1, W2, W9, W13, and W14) and Diyala (W19) was predominately in the particulate phase. This may be related to the variation in speciation of W in source materials [51]. Total manganese concentrations in the Tigris (Figure 4, supplemental material) are within the permissible levels (Table 1), but Diyala concentrations exceeded the USEPA allowable limits (Table 2). The particle partitioning of Mn was extremely variable between study sites (Figure 4—supplemental material), with many showing Mn in the particulate phase and others in the soluble phase. As was observed with many other particle reactive metals, sites W14 (53 *μ*g/L) and W19 (82.2 *μ*g/L) at Diyala River were likely influenced by the industrial discharges near these two sites [52]. With the exception of one site, Tin concentrations in the Tigris and Diyala Rivers are within the acceptable limits established by WHO and USEPA. Significant contamination is evident at site W7 where the Tin concentration of 3.04 *μ*g/L is well in excess of the permissible limits set by WHO. This site is downstream of a hospital discharge located near the Al-Sarrafia Bridge. However, this site does not influence the Tigris further downstream, as background levels return at the next site. There appears to be a shift from particle phase to dissolved phase Sn between sites W9 and W10 (Figure 4, supplemental material). Vanadium's physical speciation is dominated by dissolved species with relatively uniform concentrations observed along the Tigris transect (Figure 4). Like many of the other metals, sites W14 (Tigris) and W19 (Diyala) have elevated particulate levels (Figure 4, supplemental material). The dissolved speciation of vanadium in oxic surface waters is dominated by the oxyanion species of V(V) [53]. Silver concentrations in the Tigris are consistently low and below WHO and USEPA permissible levels (Table 1). Silver partitions strongly to particulate phases, and this is evident in the Tigris/Diyala data where particulate forms dominate the speciation of silver. Dissolved Ag is commonly associated with DOC or sulfide species [54], while particulate Ag associates with POC and iron-manganese and oxyhydroxide/sulfide phases [55]. Wastewater treatment plants are the dominant source of silver to surface waters. The Ag data from site W19 where the discharge of the treatment plant influences the Diyala is consistent with wastewater treatment plant impacts on surface water (Figure 4, supplemental material).

In general, there is no significant impact of the Diyala on the Tigris River. Most of the metals concentrations stay consistent, including sites south of the Tigris/Diyala convergence. (Figures 4 and 5, Figures  2,  3, and  4 in supplemental materials). 

### 4.4. Partition Coefficients of the Metals across the Tigris River

 The distribution of metals at equilibrium between the particulate and dissolved forms is the particle-partition coefficient *K*_*d*_ or *K*_*p*_ (L/Kg), that is, the ratio of particulate metal (*μ*g/kg) over dissolved metal (*μ*g/L). The partition coefficient *K*_*p*_ is not measured directly, rather it is calculated from measured values of adsorbed metal per unit adsorbent, divided by the concentration of dissolved metal [56]:

(1) $$\begin{array}{c} K_{p}\;\;\left(\text{L}/\text{Kg}\right)=\frac{\left[\text{SPM}\right]\;\;\left(\mu \text{g}/\text{kg}\right)}{\left[W\right]\;\;\left(\mu \text{g}/\text{L}\right)}, \end{array}$$

where [SPM] is the concentration of metal in the suspended particulate matter (the particulate form of the metal) and [*W*] represents the concentration of the metal in water (the dissolved form of the metal).

A partition coefficient may be derived as a function of TSS and other factors such as pH and salinity; thus the partition coefficient can be derived from a ratio of the particulate-sorbed and dissolved metal species multiplied by the adsorbent concentration:

(2) $$\begin{array}{c} K_{p}\;\;\left(\text{L}/\text{Kg}\right)=\left[\frac{\left[\text{SPM}\right]\;\;\left(\mu \text{g}/\text{kg}\right)}{\left[W\right]\;\;\left(\mu \text{g}/\text{L}\right)}\right]*\text{TSS}\;\;\left(\text{mg}/\text{L}\right)*10^{-6}\;\frac{\text{kg}}{\text{mg}}. \end{array}$$

TSS was used throughout this study as a measure of metal binding sites, and (2) was used to calculate the partition coefficient of the 49 metals measured in this study. Measured dissolved and particulate metals, along with the TSS, have been used to calculate the particle partition coefficients *K*_*p*_ of the 49 metals in the Tigris, Diyala, and the meeting point of the two rivers. Partition coefficients provide us with empirical information on the combined effect of heterogeneous reactions on the solid-solution distribution of an element. Consequently, high *K*_*p*_ values indicate an affinity of an element to be associated and transported with the solid phase. Conversely, a metal that tends to be in ionic form or dissolved form has a low *K*_*p*_ value. The *K*_*p*_ values for the 49 metals are tabulated in Table  1 in the supplemental materials. As shown in Figures  2,  3, and  4 in the supplemental materials, metals with a large particulate fraction exhibit high *K*_*p*_ values. We observe a decrease in *K*_*p*_ with higher TSS concentrations, suggesting the bulk of the higher TSS material is not effective at sequestering trace elements rather it is acting as a dilutional mechanism. For example, the high value of TSS (~270 mg/L) in the Diyala River has affected the partition coefficient values of all the metals, and although most of the metals are found in the particulate form, their partition coefficients are low due to the high TSS value from the Diyala River (Table 1 in the supplemental materials).

In Figure 5 of the supplemental materials we present a ranking of the mean *K*_*p*_ values (L/Kg), from highest to lowest for each element. High partition coefficients elements such as Ti, Th, Fe, Al, Pb, Cr, Co, Mn, and the REE are generally consistent with their expected geochemical properties (i.e., high particle affinity and insolubility) [39, 57]. pH, DO, POC, DOC, and SPM levels are the major chemical properties affecting the affinity of elements to particles. Most of the metals are less soluble at high pH and are removed from water column in high pH environments [43]. DO has a large impact on the distribution of the metals by changing the oxidation state of the elements [58], while some elements are strongly affected by POC, DOC, and SPM [59]. 

Iraq is subject to frequent dust and sand storms particularly in the summer time (May to September), which can add large quantities of particles to surface waters (Tigris, Euphrates, and Arab sea) [60]. Furthermore, the low discharge of the Tigris River may magnify the impact of these storms on particle concentrations in the rivers. The influence of these aeolian inputs on trace element levels and particle partitioning is not clear, but it is unlikely that these inputs are effective metal sorbers; thus their role may be a dilutive effect on *K*_*p*_ values.

### 4.5. Comparison of the Tigris with the Major Rivers in the World

 To provide context for the metal levels measured in this study, we compared the Tigris elemental data with that from major rivers in the world (Nile, Amazon, Mississippi, Ganga, and Seine River), which pass through large cities. A comparison of seven metals (Cd, Cu, Fe, Pb, Mn, Ni, and Zn) is presented in Figure 2 in the supplemental materials section. In general, the metal levels in the Tigris are similar to those in other rivers [61–64] as shown in Table 2 in the supplemental materials section, and very similar to the Seine River which passes through Paris France. Levels of metals in the Amazon River (passes through Manaus city) are lower than the Tigris [65], and metal concentrations in the Nile River (passes through Cairo city) and the Ganga River (passes through Mirzapur city) were substantially higher than the Tigris. All the metals in the Mississippi River which passes through Saint Louis exhibit low concentrations except for lead (10.7 *μ*g/L) which is approximately 20 times higher than in the Tigris River (0.479 *μ*g/L).

## 5. Conclusion

 This study presents the first comprehensive evaluation of the distribution of a large suite of metals across the Tigris River in Baghdad City. The concentrations and particle partitioning of 49 metals were measured at 17 sites along the river to establish baseline data and to further our understanding of the behavior of dissolved and particulate metals in the river. We can conclude that the Tigris River in Baghdad city is not a highly polluted river with regard to toxic heavy metals. Most of the metals that have negative health effects were within the permissible limits of WHO and USEPA for the Tigris River, except for Al, Fe, Sr, and Sn at sites close to factories and hospitals discharges. And most of the metals had no significant increase or change across the river transect, except for some metals that are affected by the facilities discharges along the river. High particulate metals were observed at site W3, which is near the treatment plant discharge in Al-Tajee district north of Baghdad city. There was high dissolved-Sn and W at site W7, which was downstream of the hospital discharge near Al-Sarrafia Bridge. In comparison with other major world rivers, the Tigris has relatively low and acceptable heavy metals concentrations. Metals concentrations in the Tigris are lower than in both Nile (Cairo city) and Ganga (Mirzapur city) rivers and comparable concentrations to the Seine River (Paris). Metals concentrations in the Amazon (Manaus) and Mississippi (Saint Louis) rivers are lower than the Tigris with the exception of high concentrations of lead in the Mississippi River. High concentrations of multiple metals were observed in the Diyala River, contributing significantly to metal levels in the Tigris, downstream from their confluence in South Baghdad city. However, the Diyala River sites (W19, W20) are near a wastewater treatment plant, particularly site W19 which is downstream of the discharge of the treatment plant. Finally, the metals that are first row transition metals and/or elements with high oxidation states “readily hydrolyzed” (e.g., Ti, Th, Fe, Al, Pb, Cr, Co, and Mn) are found to have the highest particle partition coefficients in the Tigris River. Further analysis such as speciation analysis of metals at the polluted sites needs to be performed to identify the mobility, bioavailability, and the toxicity of these metals in river water.

## Supplementary Material

Figure 1: Box plots of the total REE concentrations across Tigris River
Figure 2: Distribution of Na, k, P, and Al with the partition coefficients in Tigris (T), Diyala (D) and the meeting point (TD) of the two rivers
Figure 3: Distribution of Th, Tl, Mo, and W with the partition coefficients in Tigris (T), Diyala (D), and the meeting point (TD) of the two rivers
Figure 4: Distribution of Mn, Sn, V, and Ag with the partition coefficients in Tigris (T), Diyala (D), and the meeting point (TD) of the two rivers
Figure 5: The average values of the partition coefficients (Kp) with the uncertainties of the metals across the Tigris River
Table 1: Total metals concentrations (Mean) ± Uncertainties with partitioning coefficients for Tigris and Diyala Rivers
Table 2: The comparison of Tigris with the major rivers in the world

## Figures and Tables

**Table 1:** Toxic metals (min–max) *μ*g/L in Tigris River, with the permissible limits.

		USEPA limits^+^		WHO limits^++^		
Element	(Min–max) *μ*g/L	*μ*g/L		*μ*g/L		WA. *μ*g/L^†^
		MCL^a^	SDWR^b^	DWGV^c^	S, N, F, D (W)^d^	
Aluminum (Al)	347–2,220	—	50–200	100 (LF), 200 (SF)*	—	40
Antimony (Sb)	0.099–0.202	6	—	20	0.1–0.2 S	—
Arsenic (As)	0.99–1.76	10	—	10	1-2 N	1
Barium (Ba)	37.9–61.9	2000	—	700	100 D	—
Boron (B)	75–122	—	—	2400	500 D	30
Cadmium (Cd)	0.016–0.039	5	5	3	1 D	0.001
Chromium (Cr)	1.48–9.03	100	—	50	—	0.1
Copper (Cu)	0.7–12.7	1300	1000	2000	50–30,000 D	1.4
Iron (Fe)	326–2,220	—	300	500–50,000	—	50
Lead (Pb)	0.17–1.36	15	—	10	5 D	0.04
Manganese (Mn)	11.4–61.7	—	50	400	1–200 F	10
Molybdenum (Mo)	2.58–4.56	—	—	70	—	0.8
Nickel (Ni)	2.58–12.7	—	—	70	20 D	0.4
Silver (Ag)	0.002–0.026	—	100	5	50 D**	—
Strontium (Sr)	528–1304	—	—	—	—	100
Thallium (Tl)	0.011–0.026	20	—	—	—	—
Tin (Sn)	0.012–0.181	—	—	0.006–0.01	1-2***	—
Uranium (U)	0.7–1.2	30	—	15	1 D	—
Zinc (Zn)	1.19–7.49	—	5000	10–50	300****	0.2

USEPA limits: ^+^from [22]. WHO limits: ^++^from [23–25]. MCL^a^: maximum contaminant level; SDWR^b^: secondary drinking water regulations; DWGV^c^: drinking water guidelines value; S, N, F, D (W)^d^: surface, natural, fresh, drinking water; *(LF): large water treatment facility, (SF): small water treatment facility; **drinking water treated with Ag for disinfection; ***value is exceptional; ****unacceptable. WA^†^: world average of trace elements in unpolluted rivers.

**Table 2:** Total toxic metals concentrations (mean) ± uncertainties with partitioning coefficients for Tigris and Diyala Rivers.

Element	Tigris		Diyala	
	Total conc. *μ*g/L	*K*_*p*_ , L/Kg	Total conc. *μ*g/L	*K*_*p*_ , L/Kg
	(Mean) ± uncertainty	(Mean ∗ 10^6^)	(Mean) ± uncertainty	(Mean ∗ 10^3^)
	*n* = 17	*n* = 17	*n* = 2	*n* = 2
Aluminum (Al)	902 ± 139	13.3	2,700 ± 61	618
Antimony (Sb)	0.136 ± 0.032	1.2	0.41 ± 0.02	7.92
Arsenic (As)	1.35 ± 0.86	1.24	2.66 ± 0.41	1.98
Barium (Ba)	50.4 ± 2.9	1.59	63.4 ± 0.9	0.652
Boron (B)	98.7 ± 3.6	1.14	561 ± 5	0.774
Cadmium (Cd)	0.024 ± 0.018	2.03	0.112 ± 0.009	16.1
Chromium (Cr)	3.67 ± 4.24	4.65	9.3 ± 0.2	102
Copper (Cu)	2.11 ± 0.45	2.11	7.39 ± 0.39	326
Iron (Fe)	835 ± 85	15.2	3,130 ± 107	299
Lead (Pb)	0.479 ± 0.043	4.49	8.7 ± 0.1	357
Manganese (Mn)	27.9 ± 2.2	4.17	190 ± 3	1.68
Molybdenum (Mo)	3.48 ± 0.26	1.11	10.5 ± 0.3	14.0
Nickel (Ni)	5.11 ± 0.94	3.1	13.7 ± 0.5	20.7
Silver (Ag)	0.009 ± 0.009	0.439	0.14 ± 0.01	725
Strontium (Sr)	822 ± 49	1.12	2,570 ± 47	ND
Thallium (Tl)	0.016 ± 0.006	1.71	0.015 ± 0.003	104
Tin (Sn)	0.092 ± 0.033	0.191	0.287 ± 0.025	10.8
Uranium (U)	0.885 ± 0.043	1.63	2.8 ± 0.02	2.26
Zinc (Zn)	2.78 ± 0.93	346	42.9 ± 0.7	139

ND: not determined.

## Abbreviations

DO: Dissolved oxygen
DOC: Dissolved organic compounds
GPS: Global positioning system
HEPA: Hygiene Environmental Protection Agency
IDWQS: Iraqi drinking water quality standard
LDPE: Low density polyethylene
POC: Particulate organic compounds
QA: Quality assurance
REE: Rear Earth Elements
SF-ICP-MS: Sector field-inductively coupled plasma-mass spectrometer
SP.C: Specific conductance
SPM: Suspended particulate matter
SRM: Standard reference material
TSS: Total suspended particles
TSS/SPM: Total suspended particle or suspended particulate matter
USEPA: United States Environmental Protection Agency
WA: World average of trace elements in unpolluted rivers
WHO: World Health Organization.
