# Supplementary material for: The Distribution between the Dissolved and the Particulate Forms of 49 Metals across the Tigris River, Baghdad, Iraq
Source: ScientificWorldJournal. 2012 Nov 28;2012:246059. doi: 10.1100/2012/246059 (PMC3523588; doi:10.1100/2012/246059)
Supplement: Supplementary file 1 — Figure 1: Box plots of the total REE concentrations across Tigris River Figure 2: Distribution of Na, k, P, and Al with the partition coefficients in Tigris (T), Diyala (D) and the meeting point (TD) of the two rivers Figure 3: Distribution of Th, Tl, Mo, and W with the partition coefficients in Tigris (T), Diyala (D), and the meeting point (TD) of the two rivers Figure 4: Distribution of Mn, Sn, V, and Ag with the partition coefficients in Tigris (T), Diyala (D), and the meeting point (TD) of the two rivers Figure 5: The average values of the partition coefficients (Kp) with the uncertainties of the metals across the Tigris River Table 1: Total metals concentrations (Mean) ± Uncertainties with partitioning coefficients for Tigris and Diyala Rivers Table 2: The comparison of Tigris with the major rivers in the world [file 246059.f1.docx]

**Supplemental Materials**


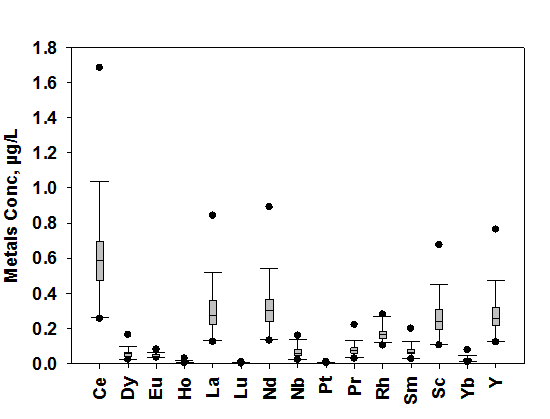


**Figure 1: Box plots of the total REE concentrations across Tigris River**

**Table 1: Total metals concentrations (Mean) ± Uncertainties with partitioning coefficients for Tigris and Diyala Rivers**

| **Element** | **Tigris** | | **Diyala** | | **Element** | **Tigris** | | **Diyala** | |
| --- | --- | --- | --- | --- | --- | --- | --- | --- | --- |
|  | **Total Conc. µg/L** | **Kp, L/Kg** | **Total Conc. µg/L** | **Kp, L/Kg** |  | **Total Conc. µg/L** | **Kp, L/Kg** | **Total Conc. µg/L** | **Kp, L/Kg** |
|  | **(Mean) ±uncertainty**  **n=17** | **(Mean*10^6^)**  **n=17** | **(Mean) ±uncertainty**  **n=2** | **(Mean*10^3^)**  **n=2** |  | **(Mean) ±uncertainty**  **n=17** | **(Mean*10^6^)**  **n=17** | **(Mean) ±uncertainty**  **n=2** | **(Mean*10^3^)**  **n=2** |
| **Aluminum(Al)** | 902±139 | 13.3 | 2,700±61 | 618 | **Niobium(Nb)** | 0.07±0.11 | 15.5 | 0.139±0.009 | 65.8 |
| **Antimony(Sb)** | 0.136±0.032 | 1.2 | 0.41±0.02 | 7.92 | **Palladium(Pd)** | 1.9±0.2 | 1.25 | 5.68±0.09 | N.D |
| **Arsenic(As)** | 1.35±0.86 | 1.24 | 2.66±0.41 | 1.98 | **Phosphorous(P)** | 35.7±5.9 | 2.97 | 2,140±60 | N.D |
| **Barium(Ba)** | 50.4±2.9 | 1.59 | 63.4±0.9 | 0.652 | **Platinum(Pt)** | 0.007±0.005 | 0.118 | 0.007±0.002 | 2.68 |
| **Boron(B)** | 98.8±3.6 | 1.14 | 561±5 | 0.774 | **Potassium(K)** | 2,240±228 | 1.14 | 13,800±482 | 1,140 |
| **Cadmium(Cd)** | 0.024±0.017 | 2.03 | 0.112±0.009 | 16.1 | **Praseodymium(Pr)** | 0.08±0.01 | 13.2 | 0.277±0.004 | 1,490 |
| **Calcium(Ca)** | 72,800±4,857 | 1.28 | 145,000±2,684 | N.D | **Rhodium(Rh)** | 0.172±0.023 | 1.22 | 0.493±0.016 | N.D |
| **Cerium(Ce)** | 0.632±0.079 | 11 | 2.16±0.04 | 2,000 | **Rubidium(Rb)** | 1.55±0.17 | 1.92 | 9.87±0.16 | 2.37 |
| **Cesium(Cs)** | 0.076±0.009 | 10.7 | 0.197±0.006 | 17.5 | **Samarium(Sm)** | 0.074±0.025 | 10.3 | 0.26±0.02 | 922 |
| **Chromium(Cr)** | 3.67±4.24 | 4.65 | 9.303±0.172 | 102 | **Scandium(Sc)** | 0.261±0.097 | 0.277 | 0.709±0.023 | 292 |
| **Cobalt(Co)** | 0.571±0.174 | 4.09 | 2.06±0.07 | 21.5 | **Silver(Ag)** | 0.009±0.009 | 0.439 | 0.14±0.01 | 725 |
| **Copper(Cu)** | 2.11±0.45 | 2.11 | 7.39±0.39 | 326 | **Sodium(Na)** | 53,700±2,370 | 1.15 | 398,000±5,650 | 0.841 |
| **Dysprosium(Dy)** | 0.058±0.026 | 8.99 | 0.211±0.011 | 1,020 | **Strontium(Sr)** | 822±49 | N.D | 2,570±47 | N.D |
| **Europium(Eu)** | 0.049±0.011 | 2.1 | 0.101±0.005 | 4.89 | **Sulfur(S)** | 61,600±5,900 | 1.3 | 293,000±7,320 | N.D |
| **Holmium(Ho)** | 0.01±0.01 | 8.07 | 0.042±0.002 | 566 | **Thallium(Tl)** | 0.016±0.006 | 1.71 | 0.015±0.003 | 104 |
| **Iron(Fe)** | 835±85 | 15.2 | 3,130±107 | 299 | **Thorium(Th)** | 0.091±0.004 | 35.5 | 0.198±0.008 | 686 |
| **Lanthanum(La)** | 0.309±0.085 | 9.75 | 1.05±0.03 | 1,210 | **Tin(Sn)** | 0.092±0.019 | 0.191 | 0.287±0.016 | 10.8 |
| **Lead(Pb)** | 0.479±0.043 | 4.49 | 8.7±0.1 | 357 | **Titanium(Ti)** | 26.2±12.7 | 40.1 | 68.2±2.3 | 400.49 |
| **Lithium(Li)** | 6.18±0.41 | 1.3 | 24.3±0.2 | 2.73 | **Tungsten(W)** | 0.078±0.114 | 0.888 | 0.102±0.035 | 0.684 |
| **Lutetium(Lu)** | 0.004±0.002 | 7.63 | 0.013±0.001 | 228 | **Uranium(U)** | 0.886±0.043 | 1.63 | 2.8±0.02 | 2.26 |
| **Magnesium(Mg)** | 21,400±1,579 | 1.02 | 112,000±3,354 | 2.46 | **Vanadium(V)** | 4.65±0.56 | 1.79 | 7.79±0.18 | 19.5 |
| **Manganese(Mn)** | 27.9±2.2 | 4.17 | 190±3 | 1.68 | **Ytterbium(Yb)** | 0.026±0.023 | 10.6 | 0.085±0.013 | 486 |
| **Molybdenum(Mo)** | 3.48±0.26 | 1.11 | 10.5±0.3 | 14 | **Yttrium(Y)** | 0.288±0.042 | 5.67 | 1.097±0.047 | 71.5 |
| **Neodymium(Nd)** | 0.327±0.046 | 9.83 | 1.15±0.05 | 938 | **Zinc(Zn)** | 2.78±0.93 | 0.346 | 43.9±0.7 | 139 |
| **Nickel(Ni)** | 5.11±0.94 | 3.1 | 13.7±0.5 | 20.7 |  |  |  |  |  |

N.D: Not Determined


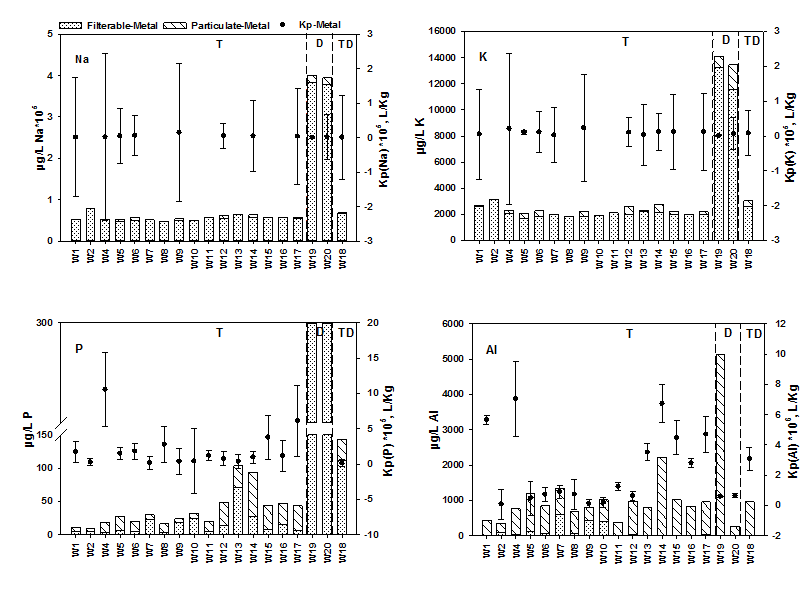

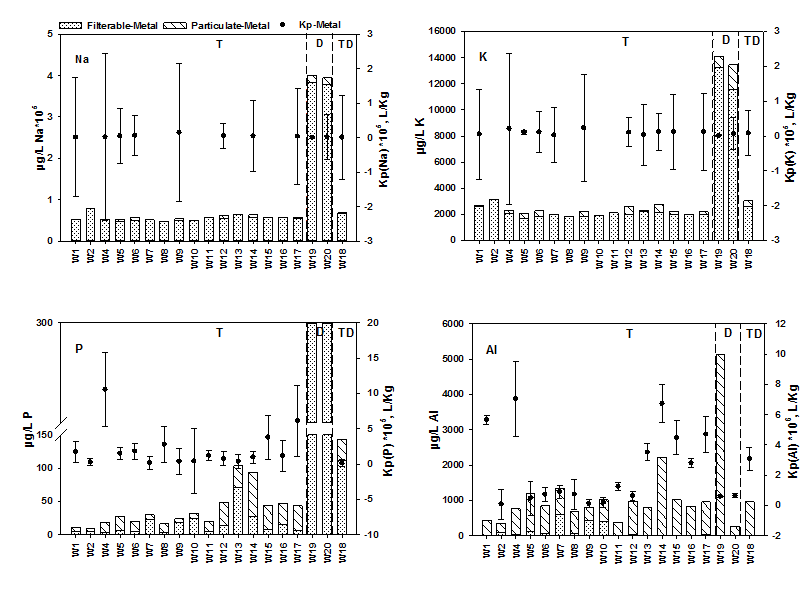

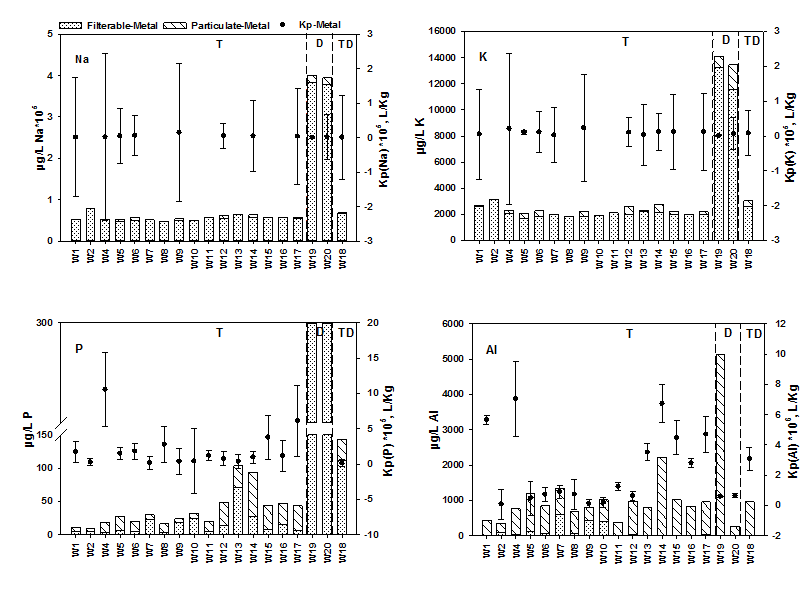

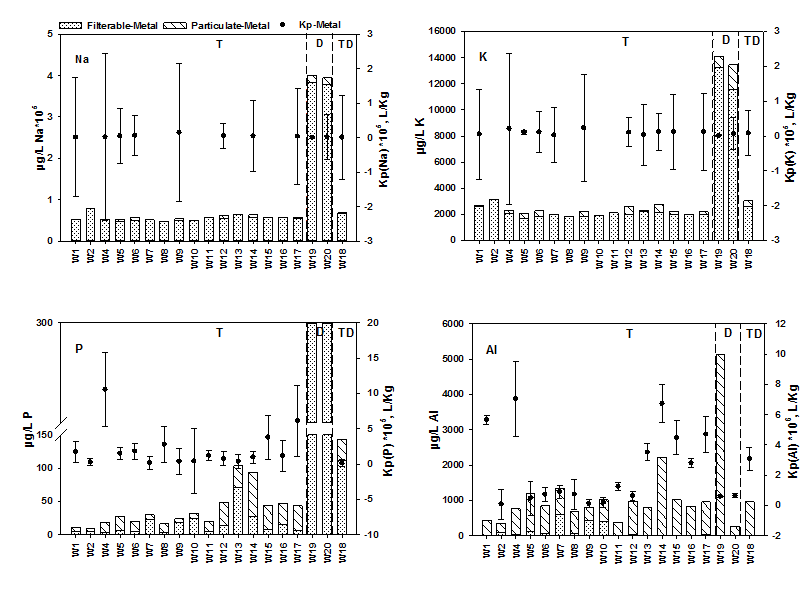


**Figure 2: Distribution of Na, k, P, and Al with the partition coefficients in Tigris (T), Diyala (D) and the meeting point (TD) of the two rivers**


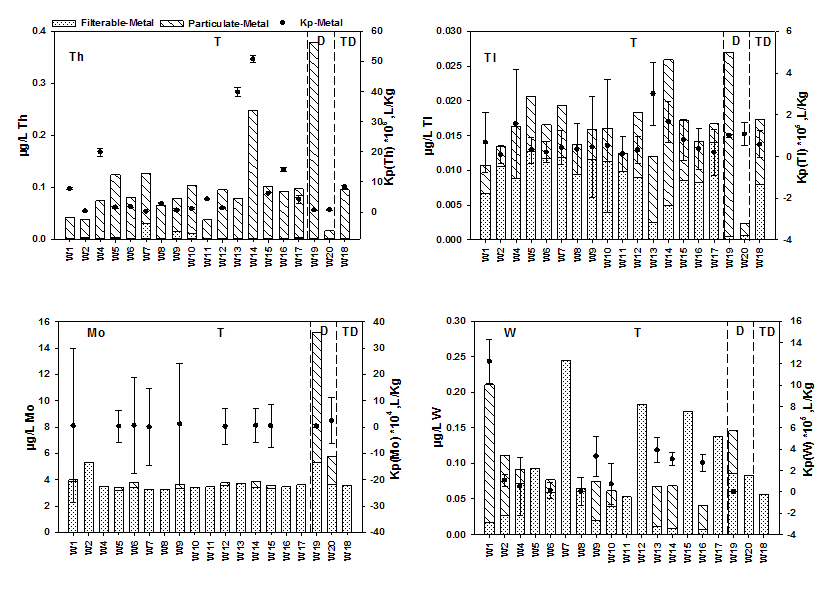

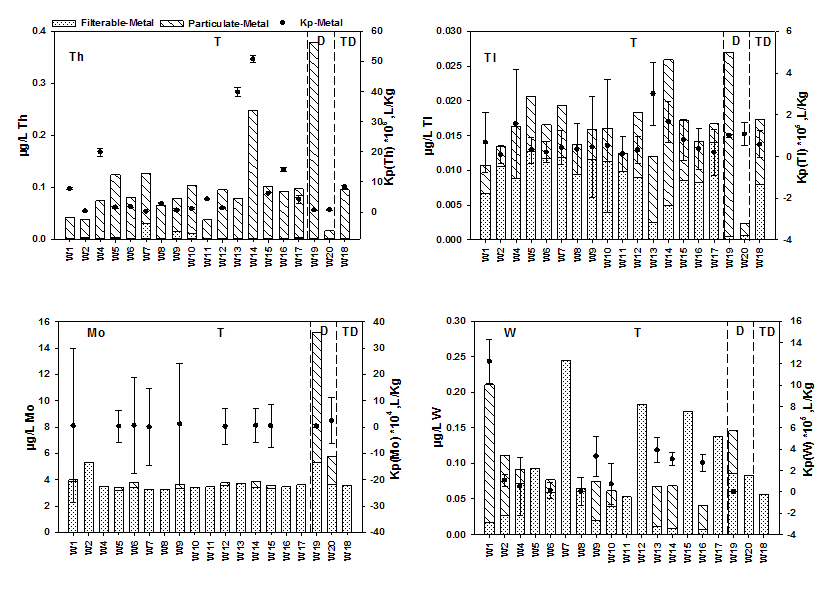


**Figure 3: Distribution of Th, Tl, Mo, and W with the partition coefficients in Tigris (T), Diyala (D), and the**

**meeting point (TD) of the two rivers**


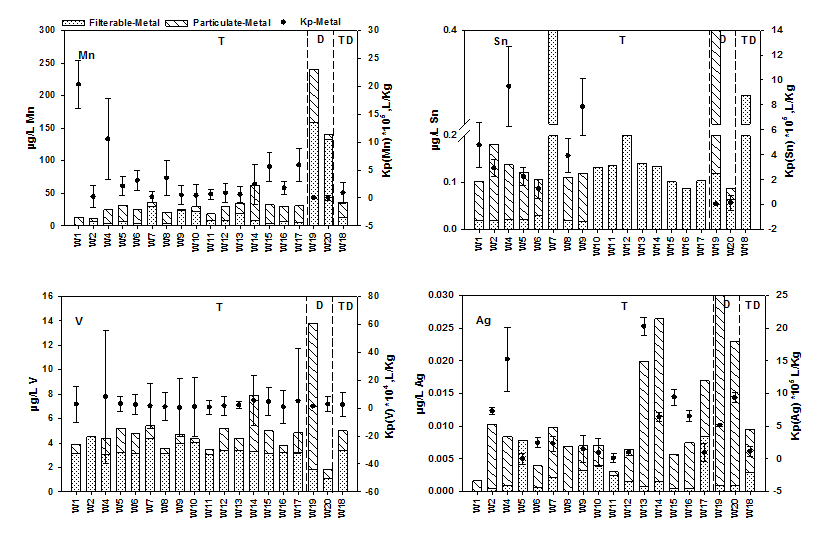


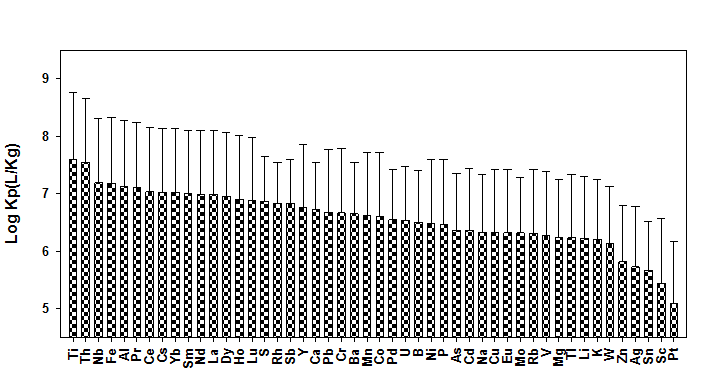
**Figure 4: Distribution of Mn, Sn, V, and Ag with the partition coefficients in Tigris (T), Diyala (D), and the meeting point (TD) of the two rivers**

**Figure 5: The average values of the partition coefficients (Kp) with the uncertainties of the metals across the Tigris River**

**Table 2: The comparison of Tigris with the major rivers in the world**

[ ]^*^: River average discharge in m^3^/s. ( )^†^: Population of the City.

| **Elements**  **µg/L** | **Tigris River**  **[1,014]^*^** | **Nile River**  **[2,830]** | **Amazon River**  **[209,000]** | **Mississippi River**  **[16,792]** | **Ganga River**  **[12,500]** | **Seine River**  **[776]** |
| --- | --- | --- | --- | --- | --- | --- |
|  | **Baghdad(7,216,000)**^†^ | **Cairo(6,760,000)** | **Manaus(1,710,000)** | **Saint Louis(319,294)** | **Mirzapur(205,264)** | **Paris(2,211,297)** |
|  | **Iraq** | **Egypt** | **Brazil** | **North America** | **India** | **France** |
| **Cd** | 0.024 | - | 0.017 | - | 13.4-32.7 | 0.031 |
| **Cu** | 2.11 | 27.8 | 1.39 | 2.14 | 38-158 | 2.23 |
| **Fe** | 835 | 1,230 | - | - | 19.8-72.8 | - |
| **Pb** | 0.479 | 15.9 | - | 10.7 | 34.3-186 | 0.354 |
| **Mn** | 27.9 | 130 | 5.54 | 0.93 | 34.3-106 | 6.26 |
| **Ni** | 5.11 | - | 0.176 | 1.52 | 67-176 | - |
| **Zn** | 2.78 | 35.8 | 0.719 | 0.327 | 94-424 | - |
